# Spontaneous resolution of a traumatic cataract in a patient with an open-globe ocular injury: a case report

**DOI:** 10.1186/s12886-020-01555-1

**Published:** 2020-07-13

**Authors:** Yu-ting Zhang, Li-qun Du, Mei Liu, Jing Zhu

**Affiliations:** Department of Ophthalmology, Qilu Hospital of Shandong University, 107#, Wenhua Xi Road, Jinan, Shandong 250012 PR China

**Keywords:** Pediatric traumatic cataract, open-globe injury, conservative cataract management, spontaneous resolution, Posterior cortical cataract, Case report

## Abstract

**Background:**

We report a case of spontaneous resolution of a traumatic cataract in a patient with an open-globe ocular injury. This case highlights the importance of conservative management in these types of cases, as excellent visual outcome is possible without invasive surgical intervention.

**Case presentation:**

A 13-year-old boy presented with a corneal laceration in the left eye caused by a neuter pen. He underwent emergency repair of the corneal laceration under general anesthesia, and at 3 days post-op, a dense posterior cortical cataract was observed. Based on the patient’s age and normal visual development, in addition to preserving accommodative potential, the patient received conservative management and follow-up. Interestingly, the cataract spontaneously resolved over the following 9 months and the corrected distance visual acuity in the injured eye was restored from finger counting at 50 cm, to 20/25 + 3.

**Conclusions:**

To optimize treatment in pediatric traumatic cataract, several critical factors such as age, visual development and the preservation of accommodative potential, need to be comprehensively considered. Conservative management with lens preservation is important to consider in young, traumatic cataract patients where invasive surgical intervention may not be required.

## Background

Spontaneous resolution of posterior cortical cataract (PCC) has been reported previously in cases where the cataract is caused by uncontrolled acute hyperglycemia [1] or transient feathering of the lens after intraocular gas tamponade [2]. In the case of traumatic cataracts, spontaneous reversal of PCC mainly occurs with mild or limited ocular injury, such as mild blunt contusion or small intralenticular foreign body [3, 4]. In severe open-globe ocular injury cases, spontaneous resolution of extensive dense PCC is rare [5], and cataract extraction, usually in combination with intraocular lens implantation, is required for vision correction [6, 7].

Here we report an unusual, spontaneous resolution of a dense PCC in a patient with severe open-globe ocular injury. Surprisingly, the patient achieved excellent visual outcome without surgical intervention.

## Case presentation

**Fig. 1:**
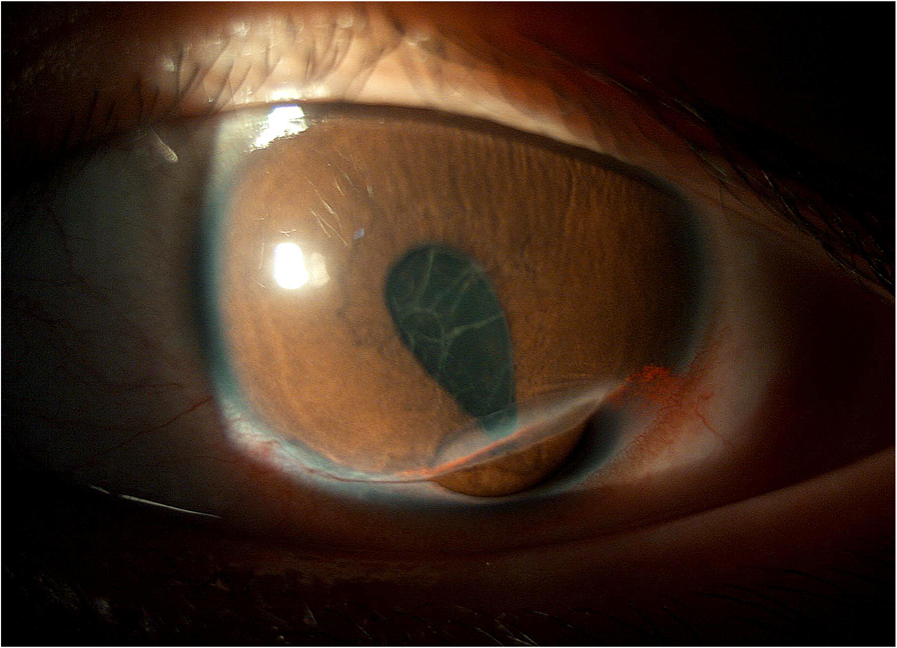
Preoperative slit lamp photograph of the patient’s left eye. There was a full-thickness corneal laceration from 4 to 8 o’clock with iris prolapse and the anterior surface of the lens was covered with an exudative membrane

**Fig. 2:**
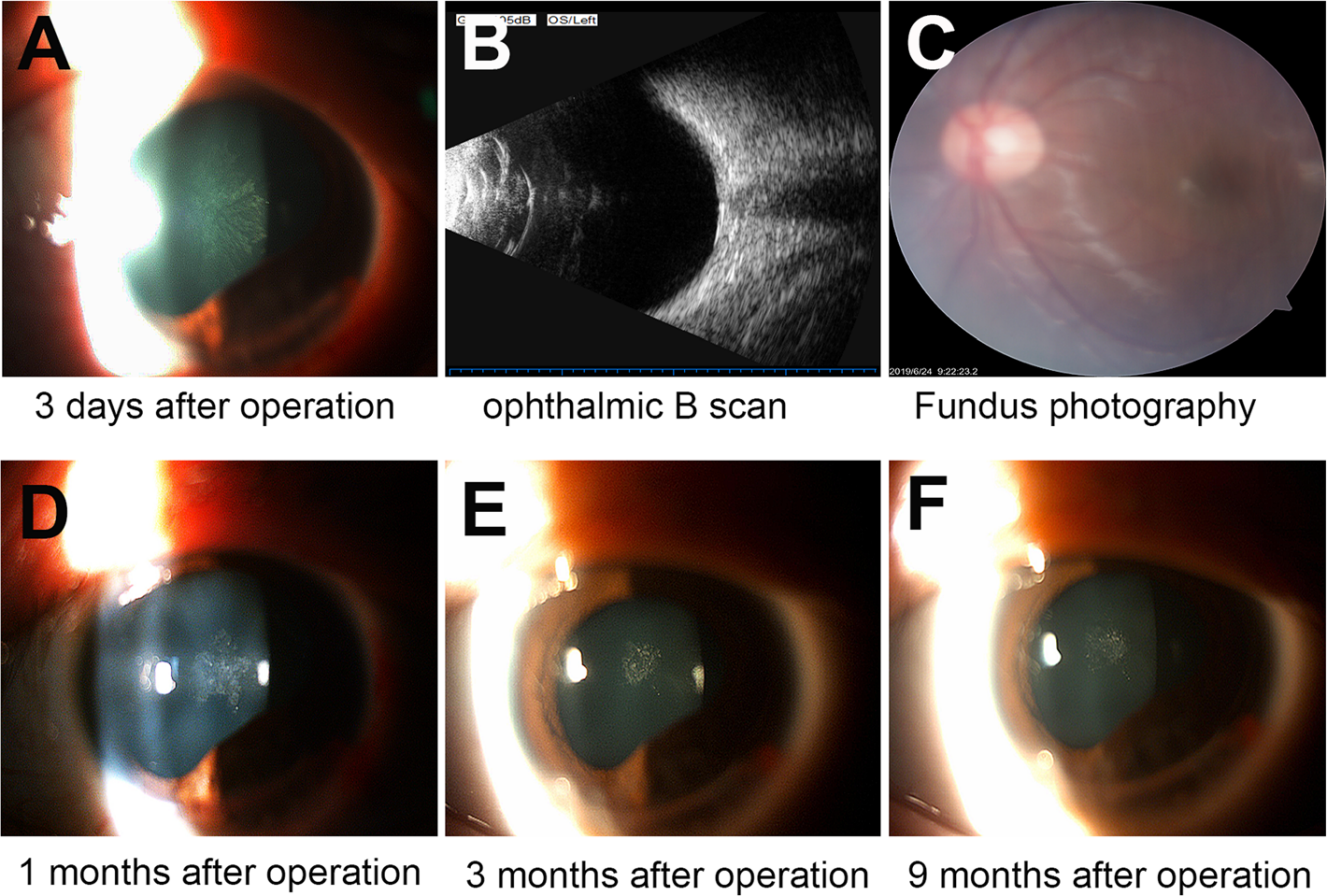
The change of PCC at different time during the spontaneous resolution. **a** 3 days after surgery, a rosette-shaped PCC was observed, **b** Ophthalmic B scan 6 days after surgery, **c** Fundus photography 6 days after surgery, **d**, **e** The size of the PCC began to decrease from 1 month after surgery, **f** 9 months after surgery, only a faint imprint of the original cataract remained

A 13-year-old boy presented to us an hour after sustaining an injury to his left eye from a red neuter pen. On examination, uncorrected visual acuity was 20/40 in the right eye and finger counting at 50 cm in the left eye. Corrected distance visual acuity (CDVA) was 20/20 with a refraction of − 1.50-2.00 × 001 in the right eye, and no improvement in the left eye. Slit-lamp examination of the left eye showed a full-thickness corneal laceration from 4 to 8 o’clock, inside the limbus, with iris prolapse. Anterior chamber depth was shallow, and an exudative membrane was noted on the anterior lens surface (Fig. 1). This patient underwent emergency repair of the corneal laceration under general anesthesia. The laceration was repaired using an interrupted suture technique with 10–0 nylon sutures, and the exudative membrane was removed using microscopic tweezers. The anterior lenticular capsule appeared intact. Post-operatively, the patient received conventional topical steroid therapy (Tobradex eyedrops 4 times a day and Tobradex eye ointment at bedtime) to reduce inflammation. Three days after surgery, a rosette-shaped PCC was observed in the left eye, while the anterior capsule and lens nucleus were still intact and transparent (Fig. 2a). The lens of the right eye was normal. Within another 3 days, the PCC increased in density and covered the whole pupillary area. The CDVA was 20/133 in the left eye (refraction of Pl-4.00 × 070). Ophthalmic B scan ultrasound examination showed increased echo of the lenticular posterior capsule with artifacts (Fig. 2b). Fundoscopic examination of the left eye were difficult to perform secondary to the cataract, however, no gross abnormalities were noted (Fig. 2c). Options for management of the cataract, including cataract surgery, were discussed with the patient’s father. Considering his age and good visual development, we agreed upon conservative observation. The patient returned for cataract follow-up bi-weekly, and waited for an appropriate time for surgery. At the one-month follow-up, we observed that the size of the PCC began to decrease (Fig. 2d, e). Nine months later, only a faint imprint of the original cataract remained and CDVA was restored to 20/25^+ 3^ with a refraction of Pl-1.50 × 070 in the left eye (Fig. 2f).

## Discussion and conclusions

Rapidly developing traumatic cataract is a common manifestation in ocular injury cases, such as intralenticular foreign body, contusive injury and open-globe ocular injury. The lens epithelium and fiber cells become damaged during injury, disrupting the integrity and permeability of the lenticular capsule. This leads to an influx of aqueous humor into the lens lamella, causing it to swell and irreversibly opacify secondary to the denaturation of lens proteins [8]. However, if the lenticular capsule damage is limited, the proliferative lens epithelium can reseal the wound without progressive cataract formation, and in rare cases, allow cataract resolution [3]. Brini A. et al. inferred that the resorption of small superficial lens opacities may occur in a pseudo-lysosomal fashion [9]. Neumayer T. et al. observed the changes and disappearance of lens vacuoles within 4 weeks in PCC patients, which might partially explain PCC spontaneous regression [10].

In open-globe injury cases, cataract formation results from direct impact, and the location of the most dense opacity commonly indicates the point of greatest impact [3, 11]. Paradoxically, in our case, cataract density was worst at the posterior cortex and not the anterior part of lens. Previous reports indicate that in blunt ocular trauma, posterior capsular rupture is more likely to appear in children and young adults [12, 13]. Wolter proposed that the absence of a sclerotic nucleus and strong zonular fibers in children and teenagers may allow the force of impact to transmit to the posterior part of lens [14]. We hypothesize that in this open-globe injury case, the PCC is secondary to indirect shock waves stemming from impact.

In young cataract patients, treatment options are more debated and require further consideration. It is known that the critical period of eye development ranges from 2 to 6 months of age, and emmetropization is generally achieved by 9 years of age [15]. To avoid amblyopia, some ophthalmologists recommend early surgical intervention for pediatric ocular traumatic cataracts, and in simple PCC cases without corneal or retinal injury, cataract extraction with intraocular lens implantation can restore excellent visual acuity [6, 7]. However, in young patients, cataract extraction also results in the loss of accommodation. For children, loss of accommodation and physiologic hyperopic reserve might affect the progress of emmetropization [16]. For teenagers, this loss of accommodation aggravates asthenopia during near work in daily life. In our case, due to the patient’s age and normal visual development, we selected the conservative approach of observation and obtained an excellent prognosis.

Our case highlights that several critical factors such as age, visual development and the preservation of accommodative potential, need to be weighed to optimize the treatment of pediatric ocular traumatic cataracts. Importantly, conservative observation may greatly benefit the young patient by avoiding unnecessary invasive surgical intervention and subsequent loss of accommodation.

## Data Availability

The datasets used and/or analysed during the current study are available from the corresponding author on reasonable request.

## Abbreviations

CDVA: Corrected distance visual acuity
PCC: Posterior cortical cataract
